# Current Progress and Challenges in Large-Scale 3D Mitochondria Instance Segmentation

**DOI:** 10.1109/TMI.2023.3320497

**Published:** 2023-09-28

**Authors:** Daniel Franco-Barranco, Zudi Lin, Won-Dong Jang, Xueying Wang, Qijia Shen, Wenjie Yin, Yutian Fan, Mingxing Li, Chang Chen, Zhiwei Xiong, Rui Xin, Hao Liu, Huai Chen, Zhili Li, Jie Zhao, Xuejin Chen, Constantin Pape, Ryan Conrad, Luke Nightingale, Joost de Folter, Martin L. Jones, Yanling Liu, Dorsa Ziaei, Stephan Huschauer, Ignacio Arganda-Carreras, Hanspeter Pfister, Donglai Wei

**Affiliations:** Department of Computer Science and Artificial IntelligenceUniversity of the Basque Country (UPV/EHU) 20018 San Sebastián Spain; Donostia International Physics Center (DIPC) 20018 San Sebastián Spain; Harvard John A. Paulson School of Engineering and Applied Sciences (SEAS)Harvard University 1812 Allston MA 02134 USA; Department of Molecular and Cellular BiologyHarvard University 1812 Cambridge MA 02138 USA; Wellcome Centre for Integrative NeuroimagingFMRIB, Nuffield Department of Clinical NeurosciencesUniversity of Oxford 6396 OX3 9DU Oxford U.K.; Department of Electronic Engineering and Information Science (EEIS)University of Science and Technology of China 12652 Anhui 230026 China; Institute of Image Processing and Pattern Recognition, Department of AutomationShanghai Jiao Tong University 12474 Shanghai 200240 China; National Engineering Laboratory for Brain-Inspired Intelligence Technology and ApplicationUniversity of Science and Technology of China 12652 Anhui 230026 China; European Molecular Biology Laboratory (EMBL) 69117 Heidelberg Germany; Institute for Computer Science, Georg-August-Universität Göttingen Göttingen Germany; Center for Molecular Microscopy, Center for Cancer ResearchNational Cancer Institute, National Institutes of Health Bethesda MD 20892 USA; Cancer Research Technology ProgramFrederick National Laboratory for Cancer Research 437329 Frederick MD 21701 USA; Francis Crick Institute 376570 NW1 1AT London U.K.; Advanced Biomedical Computational Science GroupFrederick National Laboratory for Cancer Research 437329 Frederick MD 21701 USA; resides in 8400 Winterthur Switzerland; IKERBASQUE, Basque Foundation for Science 48009 Bilbao Spain; Biofisika Institute 48940 Leioa Spain; Computer Science DepartmentBoston College 488551 Chestnut Hill MA 02467 USA

**Keywords:** Mitochondria, electron microscopy, 3D instance segmentation, connectomics, brain

## Abstract

In this paper, we present the results of the MitoEM challenge on mitochondria 3D instance segmentation from electron microscopy images, organized in conjunction with the IEEE-ISBI 2021 conference. Our benchmark dataset consists of two large-scale 3D volumes, one from human and one from rat cortex tissue, which are 1,986 times larger than previously used datasets. At the time of paper submission, 257 participants had registered for the challenge, 14 teams had submitted their results, and six teams participated in the challenge workshop. Here, we present eight top-performing approaches from the challenge participants, along with our own baseline strategies. Posterior to the challenge, annotation errors in the ground truth were corrected without altering the final ranking. Additionally, we present a retrospective evaluation of the scoring system which revealed that: 1) challenge metric was permissive with the false positive predictions; and 2) size-based grouping of instances did not correctly categorize mitochondria of interest. Thus, we propose a new scoring system that better reflects the correctness of the segmentation results. Although several of the top methods are compared favorably to our own baselines, substantial errors remain unsolved for mitochondria with challenging morphologies. Thus, the challenge remains open for submission and automatic evaluation, with all volumes available for download.

## I. Introduction

Mitochondria are the primary energy providers for cell activities, thus essential for metabolism. Quantification of the size and geometry of mitochondria is not only crucial to basic neuroscience research, e.g., neuron type identification [1], but also informative to clinical studies, *e.g.*, bipolar disorder [2] and diabetes [3]. High-resolution imaging technologies like electron microscopy (EM) have been used to reveal their detailed 3D geometry at the nanometer level with the terabyte data scale [4]. Consequently, to enable an in-depth biological analysis, we need high-throughput and robust 3D mitochondria instance segmentation methods. Publicly accessible datasets that can exemplify the challenges are also of essential importance for understanding the empirical gain of segmentation approaches in this field.

The goal of this study is to (1) analyze the current progress in the mitochondria segmentation task based on the results of the Large-scale 3D Mitochondria Instance Segmentation challenge (MitoEM),^1^ at the IEEE International Symposium on Biomedical Imaging (ISBI) 2021, and (2) present an in-depth analysis of the state-of-the-art evaluation metrics for identifying mitochondria instance segmentation errors, that reveals the difficulties of the current approaches and can be used as a guide for the creation of the next generation mitochondria segmentation models. To the best of our knowledge, MitoEM was the first open comparison of mitochondria instance segmentation algorithms on EM volumes. Moreover, we describe the associated annotated dataset of two 3D EM image stacks at the scale of $(32.8\times 32.8\times 30 ~\mu m)$, which are freely available from the challenge website, and are two of a few large-scale 3D image volumes suitable for testing instance segmentation algorithms.^1^Challenge website: https://mitoem.grand-challenge.org

### A. Previous Works

#### 1) Mitochondria Segmentation Datasets:

The *de facto* benchmark dataset for evaluating methods of mitochondria segmentation from EM images is the EPFL Hippocampus dataset [5], referred to as the Lucchi dataset in this paper. This dataset includes two EM image volumes along with corresponding binary segmentation masks. Subsequently, Kasthuri et al. [6] provided annotation for mitochondria masks for selected regions within a 3-cylinder volume. Additionally, Casser et al. [7] improved the annotation quality for both datasets through the implementation of a consistent annotation protocol for mask boundaries. Despite these efforts, the datasets remain small in size, less than 0.3 Gigavoxels and ( $5~\mu \text{m}$)^3^ physically, which does not adequately capture the complexity of mitochondria morphology. The complete image stack measures $2048\times 1536\times 1065$ voxels, yet only approximately 35% of it was manually annotated, comprising two sub-volumes, each with dimensions of $1024\times 768\times 165$ voxels. Furthermore, the provided binary masks are not easily converted into instance segmentation masks, which are necessary for detailed biological analysis as the instances of mitochondria can be connected to each other.

#### 2) Instance Segmentation Evaluation Metrics:

The evaluation of instance segmentation results can be done at either the pixel level or the instance level. The pixel-level metrics assume high-quality ground truth instance masks and measure the correctness of the pixel grouping with a clustering-based criterion, such as the Rand index [8]. However, as dataset sizes grow, it becomes increasingly difficult to manually refine all masks for pixel-level accuracy. As a result, instance-level metrics are more commonly used for large-scale datasets. For each predicted instance mask, if its intersection-over-union (IoU) score with a ground truth mask is higher than a predefined threshold, it is considered a true positive. Similarly, predictions that fall below the IoU threshold are considered false positives, while ground truth predictions without a match with the true positive prediction are considered false negatives. For biomedical image datasets, metrics based on true positives, false positives, and false negative rates, such as accuracy are widely used in the literature [9], [10], [11]. In the case of natural 2D images, popular methods like Mask R-CNN-based approaches, typically predict the confidence for each instance detection, and the average precision (AP) metric is used to average results over different detection thresholds [12], [13]. In addition, instances are usually divided into small/medium/large groups for separate evaluations. Wei et al. [14] provided an efficient implementation of the AP metric for instances inside 3D volumes. To further break down the analysis of instance matching results, Ka et al. [15] proposed association metrics, categorizing them into *one-to-one*, *over-segmentation*, *under-segmentation*, *many-to-many*, *missing*, and *background*. In summary, the combination of these metrics and categories allows for a comprehensive evaluation of instance segmentation methods in the context of biomedical imaging applications. While these metrics are often used individually, their collective utilization provides a more thorough assessment of performance.

#### 3) Machine Learning Methods:

Despite the advances in large-scale instance segmentation for neurons from EM images [16], [17], similar efforts for mitochondria have been largely overlooked in the field. The lack of a large-scale, public dataset has led to the majority of recent mitochondria (semantic) segmentation methods being benchmarked on the Lucchi dataset [5], where mitochondria instances are small in number, simple in morphology, and relatively sparse in distribution. Even in non-public datasets [18], [19], the complexity of mitochondrial shapes is limited by the small size of the dataset and the use of non-mammalian tissue. In the field of mitochondria semantic segmentation, previous studies have employed a variety of techniques to segment the Lucchi dataset. Early works have leveraged traditional image processing and machine learning techniques [20], [21], [22], [23], while recent methods made use of 2D or 3D deep learning architectures for mitochondria segmentation [7], [24], [25], [26]. Furthermore, Liu et al. [27] proposed an instance segmentation approach by means of a modified Mask R-CNN [28], while Xiao et al. [29] achieved instance segmentation through a tracking approach. However, it remains uncertain how the performance of these methods, developed on small datasets, would extend to larger datasets (*e.g.*, $(30 ~\mu m)^{3}$ cube) for neuroscience analysis, where mitochondria exhibit more complex variations in appearance and shape.

## II. MitoEM Challenge

### A. Dataset

The basis for this challenge is our previously released large-scale 3D mitochondria instance segmentation benchmark, known as the MitoEM dataset [14]. The MitoEM dataset comprises two 3D EM image stacks, each measuring $32.8 \times 32.8 \times 30 ~\mu m$ in size, with a voxel dimension of $8 \times 8 \times 30 nm$. These image stacks originate from distinct sources, one from adult rat brain tissue (MitoEM-R) and the other from adult human brain tissue (MitoEM-H). Notably, the MitoEM dataset represents a substantial increase in scale, being approximately **1,986 times larger**,^2^ than the previous Lucchi benchmark [5]. From the 1,000 consecutive slices of each stack, ground-truth mitochondria instance labels were provided for the first 500 slices and split into training (400 slices) and validation (100 slices) subsets. The annotations of the remaining 500 slices of each volume were kept private and used as the test set. For information regarding the dataset acquisition and annotation strategy, we refer readers to Wei et al. [14].^2^Calculated by comparing the size of the MitoEM dataset with that of the Lucchi benchmark as follows: $((4096 \times 4096 \times 1000)(8 \times 8 \times 30)) / ((165 \times 1024 \times 768)(5 \times 5 \times 5)) \approx 1986$.

#### 1) Improved Annotation (V2):

**Fig. 1.**
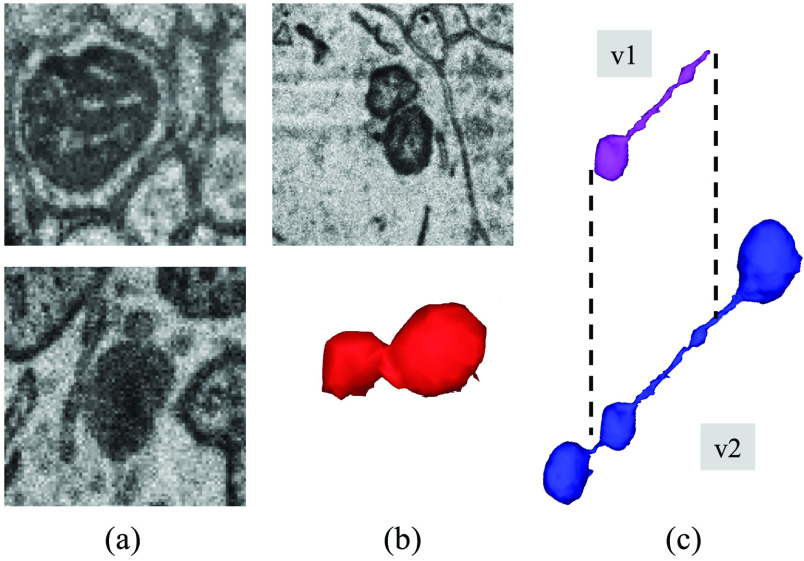
Common annotation errors in the initial MitoEM dataset [14] (v1): (a) organelles that look similar to mitochondria and where often false positives are created, (b) false merges of mitochondrion, and (c) incomplete segmentation. Those errors were fixed after another round of expert proofreading (v2).

After the initial release of the MitoEM dataset, we identified three consistent categories of annotation errors (as depicted in Fig. 1). These errors include instances of organelles with a similar dark appearance that were mistakenly labeled as mitochondria, instances of neighboring mitochondria that were falsely merged into a single mitochondrion, and instances of *mitochondria-on-a-string* (MOAS) [30] that were occasionally incomplete due to their thin microtubule connections. According to the findings reported by Zhang et al. [30], MOAS have been identified as a novel phenotype that exhibits increased prevalence during disease progression and the accumulation of mutations in both rat and human brain analyses. Therefore, accurate segmentation of this type of mitochondria, without inducing any splitting, holds significant importance in understanding its role and implications in cellular biology.

To rectify these annotation errors, we engaged the expertise of three neuroscience specialists with in-depth knowledge of EM images and mitochondria morphology. Each expert independently scrutinized the previous annotations, meticulously comparing them to the visual information depicted in the EM images. In cases where discrepancies or differences of opinion arose among the experts, collaborative discussions were held to resolve these issues and reach a consensus. Through this collaborative effort, we consolidated the necessary changes, resulting in a more accurate and reliable ground truth for the challenge.

Consequently, the number of confirmed instances in the MitoEM-H dataset was reduced from 24.5K to 19K, while in the MitoEM-R dataset, it was reduced from 14.4K to 10.8K. These revised annotations were subsequently updated and uploaded to the Grand Challenge platform in December 2021, and all participating methods were re-evaluated accordingly. Notably, despite the modifications in the annotations, the overall rankings on the leaderboard remained largely unaltered.

### B. Evaluation Metric

In our initial release of the challenge, we used the evaluation metric proposed by Wei et al. [14], which computes the AP-75 score for small/medium/large groups of instances based on the instance size. However, upon conducting an analysis of the errors in the challenge submissions, we recognized the need to make certain improvements to the evaluation metric.

#### 1) Improved Metric: From AP to Accuracy:

We found that the AP-based metrics that were originally designed for top-down instance segmentation methods, such as Mask RCNN [28], are not well-suited for our challenge. In our case, most submission methods employed a bottom-up approach for instance segmentation, in which there is no estimation of the detection confidence score for each instance. To address this issue, Wei et al. [14] approximated the confidence score with the size of the instance, which can lead to unintuitive evaluation results, as further discussed in Section IV. After careful consideration, we decided to adopt the popular accuracy metric [10] for evaluating the challenge submissions. This metric matches prediction instances with ground truth instances, providing a more intuitive evaluation of the methods’ performance.

#### 2) Improved Instance Grouping: From Volume to Cable Length:

**Fig. 2.**
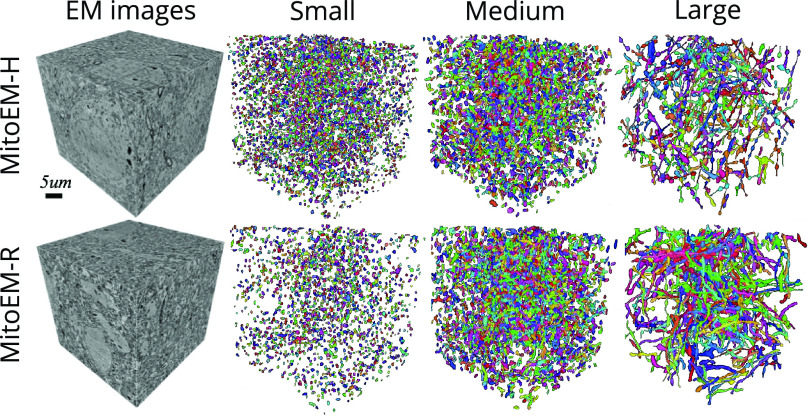
Visualization of MitoEM-H and MitoEM-R datasets splitting categories based on cable length. From left to right: original 3D EM images, and their corresponding meshes of small (length $\leq 1~\mu \text{m}$), medium ( $1~\mu \text{m} < $ length $ < 4~\mu \text{m}$), and large (length $\geq 4~\mu \text{m}$) mitochondria of human (top) and rat tissue (bottom).

In our initial release of the challenge, we used a splitting rule based on the volume to categorize mitochondria instances into small, medium, and large groups. However, we noticed that this approach was not effective for correctly categorizing complex mitochondria instances, such as the MOAS. For that reason, we opted for the cable length^3^ instead, using length thresholds of $1~\mu \text{m}$ and $4~\mu \text{m}$ to split the mitochondria into three groups: small, medium and large (as in the original MitoEM release). Under this new categorization, the number of small, medium, and large mitochondria instances are respectively: 5106, 3608, and 164 in MitoEM-H and 1292, 3832 and 524 in MitoEM-R. A visualization of the mitochondria of each new split is depicted in Fig. 2. Notably, all instances classified as MOAS are now grouped within the large category, aligning with our previous expectations. A fast inspection reveals that (1) the human tissue contains many more small mitochondria than the rat tissue, and (2) the large mitochondria from the human tissue are notably thinner than those of the rat tissue. It is important to note that these differences between human and rat tissues may not be generally extrapolated, as making such statements would require additional samples and references to establish reference ranges.^3^Cable length is defined as the skeleton length of the instance, *i.e.*, total length of all branches, taking into account each axes resolution, *e.g.*, $8\times 8\times 30$ for ( $x$, $y$, $z$) axes in MitoEM. We use Kimimaro [31] for the skeletonization.

All these changes in the evaluation became effective in July 2022 in the Grand Challenge platform. In contrast to the improved annotation, these modifications resulted in significant alterations in the leaderboard as described in Section V.

### C. Organization

The challenge was accepted to ISBI 2021 in October 2020 and officially announced in November 2020. This announcement was accompanied by the creation of a dedicated website and the preparation of an evaluation system. The two image volumes, MitoEM-R and MitoEM-H, were made immediately available to participants to enable them to begin developing their methods. Participants performed the segmentation on their own computers. The challenge was widely advertised and was open to any interested participants. A total of 257 individuals registered for the challenge and 14 teams submitted their results. For comparison, we also used two “internal submissions” corresponding to our 2D and 3D baseline methods (U2D-BC and U3D-BC, Section III-A). To lower the barrier of entry for the challenge, an initial version of the code of U3D-BC was made publicly available. The teams were also asked to submit a description of their method. Eight teams were invited to a workshop on April 13, during the ISBI 2021 conference, and to participate in the writing of this article. The winners of the challenge were announced at this workshop.

Some of the teams that participated in the challenge did not register for the conference or participate in the workshop. However, six teams did submit short papers and presented their methods. The results announced at the workshop (ranked using the original AP-75 metric) are given in Table V in the Appendix. Those results may be based on updated submissions. After the workshop, the challenge has remained open to submissions and all image volumes, as well as their ground-truth labels, are available for download. The testing labels continue to be confidential.

**TABLE I:** Overview of the MitoEM Participant Methods. Baseline Methods From the Challenge Organizers Are Also Shown (Marked With *). CE — Cross-entropy, WBCE — Weighted Binary Cross-Entropy, MSE — Mean Squared Error, WMSE — Weighted MSE, SGD — Stochastic Gradient Descent, HA — Hierarchical Agglomeration, MCWS — Marker-controlled Watershed, MWSMC — Mutex Watershed and Multicut, CC — Connected Components, HUA — Hungarian Algorithm. (Y) Reuse U3D-BC Code

Method	Available code	Model architecture	Input shape	Loss function	Optimizer	Connectivity method	Pre-/post-processing
VIDAR [32]	✓	Residual U-Net	3D	WBCE	Adam	HA	Denoising as pre-processing
IIPPR	✓ (†)	Residual U-Net	3D	BCE+Dice	Adam	MCWS	Ensemble+Blending inference, size filtering
U3D-BC*	✓	Residual U-Net	3D	BCE+Dice	SGD	MCWS	Aggressive DA
U2D-BC*	✓	U-Net	2D	BCE	SGD	MCWS	Aggressive DA+YZ-Filtering
VGG [33]		U-Net	3D	CE+Contrastive	SGD	MCWS	None
EMBL	✓	U-Net	3D	Dice	Adam	MWSMC	None
CEM-PDL [34]	✓	Panoptic-DeepLab	2D	WBCE+MSE	AdamW	HUA	CEM500K pretraining, Z-filtering...
FCI [35]	✓	U-Net	3D	Dice	Adam	MCWS	Tri-axis prediction
ABCS		U-Net	3D	Dice	Adam	CC	Ensemble
H2RNet		Hybrid-HRNet	2D	WMSE	Adam	MCWS	Morphological closing, size filtering

**TABLE II:** AP-Based, Matching-Based and Association-Based Metrics Evaluation of the Synthetic Example of Fig.4. Association Metrics are Expressed in %

		Small	Medium	Large	Total
AP-based	AP $-75\,\,\uparrow $	0.51	0.44	0.00	0.22
Matching metrics	Precision $\uparrow $	0.06	0.67	0.00	0.12
	Recall $\uparrow $	0.50	0.67	0.00	0.50
	Accuracy $\uparrow $	0.05	0.50	0.00	0.10
Association metrics	One-to-one $\uparrow $	50.0	66.7	0.00	50.0
	Missing $\downarrow $	0.00	0.00	0.00	0.00
	Over-segmentation $\downarrow $	0.00	0.00	100	16.7
	Under-segmentation $\downarrow $	50.0	33.3	0.00	33.3
	Many-to-many $\downarrow $	0.00	0.00	0.00	0.00
	Background $\downarrow $	–	–	–	65.4

**TABLE III:** Matching-Based Metrics of All Methods on the MitoEM Challenge Leaderboard. The Rankings Presented in This Table are Ordered by Overall Accuracy, Thus Differing From the Original Challenge Leaderboard, as Discussed in the Manuscript. The Baseline Methods From the Challenge Organizers (Marked With *) Are Shown but Not Included in the Ranking. The Best Scores Are Shown in Bold

Method	Rank	MitoEM-H			MitoEM-R			
		Prec. $\uparrow $	Rec. $\uparrow $	Acc. $\uparrow $	Prec. $\uparrow $	Rec. $\uparrow $	Acc. $\uparrow $	Total Acc.
IIPPR	1	0.814	0.913	0.755	0.824	0.943	0.785	0.770
VIDAR	2	0.785	0.926	0.739	0.638	0.948	0.616	0.678
U3D-BC*		0.706	0.916	0.663	0.663	0.913	0.623	0.643
EMBL	3	0.740	0.879	0.672	0.637	0.906	0.597	0.635
VGG	4	0.658	0.911	0.619	0.697	0.905	0.649	0.634
CEM-PDL	5	0.734	0.794	0.617	0.721	0.860	0.645	0.631
FCI	6	0.741	0.754	0.596	0.669	0.771	0.558	0.577
H2RNet	7	0.636	0.698	0.499	0.709	0.811	0.608	0.554
ABCS	8	0.628	0.766	0.526	0.675	0.694	0.520	0.523
U2D-BC*		0.435	0.925	0.420	0.354	0.911	0.342	0.381

**TABLE IV:** Association-Based Metrics (in %) of All Methods on the MitoEM Challenge Leaderboard. Baseline Methods From Challenge Organizers (Marked With *) Are Shown but Not Included in the Ranking. The Terms ‘Correct’, ‘Missing’, ‘Over’, ‘Under’, and ‘Many’ Represent ‘one-to-One’, ‘missing’, ‘over-segmentation’, ‘under-segmentation’, and ‘many-to-many’ Associations, Respectively. The ‘Background’ Percentage is Calculated Relative to All Predicted Instances, While the Remaining Association Values Are Calculated Relative to the Number of Ground Truth Instances and Collectively Add Up to 100%. The Best Scores are Indicated in Bold

	Accuracy Rank	MitoEM-H						MitoEM-R					
Method		Correct $\uparrow $	Missing $\downarrow $	Over $\downarrow $	Under $\downarrow $	Many $\downarrow $	Back $\downarrow $	Correct $\uparrow $	Missing $\downarrow $	Over $\downarrow $	Under $\downarrow $	Many $\downarrow $	Back $\downarrow $
IIPPR	1	93.07	1.93	1.99	2.87	0.14	8.48	94.92	0.41	2.97	1.66	0.04	5.25
VIDAR	2	92.61	1.89	3.62	1.76	0.12	11.77	93.89	0.46	3.98	1.52	0.14	24.39
U3D-BC*		91.82	1.22	4.10	2.65	0.21	17.43	90.95	0.51	4.97	4.07	0.50	15.26
EMBL	3	87.55	3.39	4.89	3.77	0.39	10.22	79.62	1.38	17.0	1.17	0.81	6.62
VGG	4	90.82	0.78	4.16	3.86	0.38	22.46	90.67	0.55	3.67	4.75	0.37	15.54
CEM-PDL	5	89.4	3.32	2.96	4.05	0.26	7.98	88.05	0.62	6.32	4.21	0.80	7.28
FCI	6	85.23	7.51	3.09	3.91	0.26	5.87	84.86	1.84	5.49	6.94	1.04	5.36
H2RNet	7	79.88	7.07	8.49	4.10	0.45	5.35	84.54	1.43	5.81	7.03	1.19	3.85
ABCS	8	76.02	2.65	3.47	16.9	0.97	18.1	67.76	0.74	2.76	25.62	3.12	10.76
U2D-BC*		91.32	0.32	6.10	2.05	0.21	49.05	88.6	0.09	7.15	3.43	0.73	52.02

**TABLE V:** The MitoEM Challenge Leaderboard as Announced at the Workshop at ISBI 2021. The Methods are Ranked According to Their AP-75 Scores, With the Highest Scores Displayed in Bold. The Rankings Presented in This Table Align With the Original Challenge Leaderboard, but Deviate From Those Presented in the Present Manuscript Due to the Modification of the Evaluation Metric. The Baseline Methods From the Challenge Organizers (Marked With *) are Displayed but Were Not Included in the Ranking

Method	AP-75 Rank	MitoEM-H				MitoEM-R				Overall
		Small	Medium	Large	All	Small	Medium	Large	All	
VIDAR	1	0.835	0.905	0.420	0.827	0.727	0.955	0.550	0.850	0.839
IIPPR	2	0.807	0.884	0.328	0.796	0.815	0.941	0.517	0.842	0.819
U3D-BC*		0.799	0.885	0.331	0.790	0.780	0.896	0.505	0.811	0.801
VGG	3	0.794	0.854	0.333	0.786	0.788	0.885	0.425	0.790	0.788
EMBL	4	0.783	0.837	0.389	0.762	0.773	0.896	0.444	0.779	0.771
U2D-BC*		0.741	0.885	0.349	0.779	0.623	0.879	0.433	0.751	0.765
CEM-PDL	5	0.642	0.742	0.249	0.644	0.730	0.834	0.194	0.674	0.659
ABCS	7	0.655	0.669	0.295	0.636	0.709	0.586	0.304	0.572	0.604
FCI	6	0.610	0.745	0.345	0.620	0.598	0.710	0.270	0.582	0.601
H2RNet	8	0.574	0.541	0.216	0.474	0.656	0.764	0.260	0.605	0.540

## III. Summary of Segmentation Methods

In this section, we present our proposed baseline methods together with the evaluated segmentation methods submitted by the eight teams who successfully completed the challenge. An overview of the principal components of each method, including our baseline methods, can be found in Table I. Detailed information about the algorithms employed by each team is provided in their respective manuscripts, which were submitted according to the MitoEM challenge policies. These manuscripts are available on the challenge webpage under the “manuscripts” tab.

### A. Open-Source Baseline Methods

**Fig. 3.**
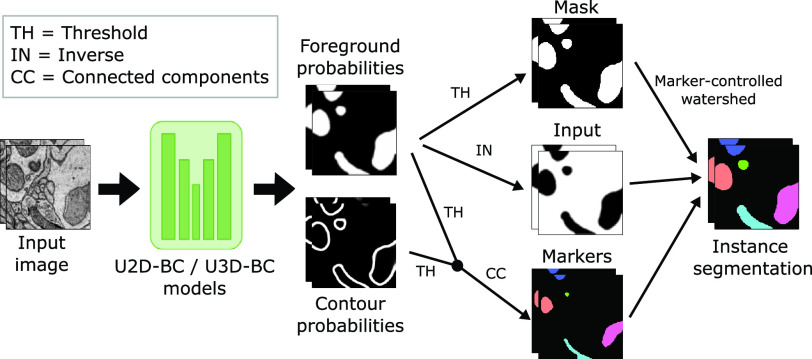
Processing pipelines of our open-source baseline methods (U2D-BC/U3D-BC). The model predicts foreground and contour probabilities that are fused to create three inputs for a marker-controlled watershed [37] to produce individual instances.

To enhance the accessibility of our challenge, we have developed and released two open-source baseline methods: U2D-BC and U3D-BC. These methods are designed to handle 2D and 3D input images, respectively. Both approaches make use of a U-Net-based architecture [36] to predict binary foreground segmentation masks and instance contours masks (referred to as *BC* in the methods’ name). Following the prediction, the two outputs are thresholded and combined. Next, a connected components operation is applied to generate distinct, non-touching mitochondria instance seeds. Subsequently, a marker-controlled watershed algorithm [37] is applied, using three key components: 1) the inverted foreground probabilities as the input image (representing the topography to be flooded), 2) the generated instance seeds as the marker image (defining starting points for the flooding process), and 3) a binarized version of the foreground probabilities as the mask image (constraining the extent of object expansion). The collective implementation of these components facilitates the creation of individual mitochondria instances (see Fig. 3 for a visual representation).

#### 1) U2d-BC:

The core architecture of the U2D-BC method consists of a 5-level U-Net. The initial level of the U-Net comprises 16 filters, which are doubled in each subsequent level. Dropout regularization is applied in each block, with the dropout rate gradually increasing from 0.1 to 0.3 (in the bottleneck layer), and then decreasing back to 0.1 in the upsampling layers. Exponential linear units (ELU) [38] are employed as activation functions. To perform upsampling in the decoder, transposed convolutions are used, following the approach proposed in [26]. The model was trained using an input size of $256\times 256$ and optimized until convergence, approximately 180 epochs, over a reduced version of the dataset (20% of training data) with stochastic gradient descent (SGD) using a fixed learning rate of 0.002. The reduced training set was created by selecting one image every four slices along the z-axis. This decision was based on the repetitive nature of the slices along this axis, where no significant 3D information would be lost in the process. The intention behind using a smaller training dataset for the U2D-BC model was to expedite the training process and provide a baseline 2D network for competitors to build upon. We further applied median filtering in the $y$- $z$ axes to improve the network output predictions. The model was implemented with BiaPy [39] and can be reproduced based on the tutorial provided by the challenge organizers.^4^^4^https://biapy.readthedocs.io/en/latest/tutorials/mitoem.html

#### 2) U3D-BC:

The U3D-BC method utilizes a 5-level residual U-Net architecture, inspired by Lee et al. [40]. The model incorporates batch normalization as a regularization technique and employs ELUs as activation functions. Transposed convolutions are used for upsampling. To account for the anisotropy of the datasets, the model is trained with an input size of $225\times 225\times 17$ for the $x$, $y$, and $z$ axes. Notably, feature maps are not downsampled or upsampled along the $z$-dimension, and each residual block consists of a combination of 2D and 3D convolutions [40]. The model was optimized for 150 epochs over the entire training data, with an initial learning rate of 0.04 and cosine learning-rate scheduling [41]. We also applied Gaussian blending and test-time augmentations (self-ensemble) to improve the prediction quality. The model was implemented with PyTorch Connectomics [42] and can be reproduced based on the tutorial provided by the challenge organizers.^5^^5^https://connectomics.readthedocs.io/en/latest/tutorials/mito.html

In comparison to our previous work [14], we made improvements to the implementation details in order to achieve superior results. Specifically, we have incorporated a number of additional data augmentation techniques, including misalignment (which simulates xy-plane displacement during data acquisition in microscopes), CutBlur [43], CutNoise, and motion-blur. These augmentations supplement the brightness, flip, elastic transform, and missing parts augmentations used in the original MitoEM paper [14]. Furthermore, we increased the probability and intensity of all augmentations to enhance the robustness of the models.

### B. Participants’ Methods

The following methods by the participant teams produced successful results that were submitted to the challenge. Notice that the method names used here may differ from the team names found on the MitoEM webpage.

• **VIDAR** (USTC)^6^ [32]: The VIDAR method proposed by the authors consists of two specialized networks: Res-UNet-R and Res-UNet-H. Both networks are designed to predict instance boundaries and semantic masks of mitochondria. Inspired by the 3D U-Net [44], both architectures incorporate residual blocks. However, the initial convolution is performed only in 2D to account for the anisotropic resolution of the input data. The encoding and decoding paths of both networks have five levels, with the number of filters per level set as follows: 28, 36, 48, 64, and 80. In the Res-UNet-R, the decoder simultaneously outputs the semantic mask and the instance boundary. On the other hand, the Res-UNet-H contains two separate decoders, one for each output. To address class imbalance, a weighted binary cross-entropy loss function is utilized during training. The authors also employ a multi-scale training strategy, dividing the training into two stages with progressively larger input images, first $256\times 256\times 32$, and then $320\times 320\times 36$. For pre-processing, the authors apply denoising using their own image restoration network [45], which is trained on patches of size $256\times 256\times 3$. During testing, coarse noisy regions in the test sets are manually selected and restored using the trained interpolation network before performing segmentation. Finally, the semantic masks and instance boundaries are used to synthesize a 3D affinity volume, which enables hierarchical agglomeration [46] for extracting individual instances.
• **IIPPR** (SJTU)^7^: The submissions of this team were based on the U3D-BC baseline method provided by the challenge organizers. The main difference from the original U3D-BC configuration is the input size of $256\times 256 \times 32$ with an overlap of $128\times 128 \times 16$. To separate touching instances, ground truth masks were eroded using a $3\times 3$ kernel, while instance contours were extracted through morphological erosion with a $7\times 7$ kernel. For MitoEM-H, they used threshold values of 0.7, 0.6, and 0.6 to extract seeds, instance contours, and foreground, respectively. For MitoEM-R, they used threshold values of 0.85, 0.6, and 0.8. Moreover, they removed all objects with fewer than 1024 voxels based on the fact that all mitochondria instances in the challenge volumes are at least 2000 voxels in size.
• **VGG** (NEL-BITA)^8^ [33]: This team used a contrastive learning [47], [48] framework, employing a representative voxel sampling strategy and a loss function that combines a voxel-wise similarity term to increase the similarity of voxels from the same class and the separability of voxels from different classes. Additionally, an inter-frame consistency term is included to enhance the sensitivity of the 3D model to changes in image content from frame to frame. The backbone network used is a classic 3D U-Net [44], which outputs binary masks and boundary maps. A marker-controlled watershed [37] algorithm is then applied to extract the final instances. Feature maps are extracted from the last two layers of the backbone decoder to capture voxel features and build positive and negative pairs based on their classes. This enables the use of contrastive learning to maximize the similarity between feature vectors of the same class while minimizing the similarity between feature vectors of different classes. Similarly, the consistency loss term is designed to enhance the feature similarity between voxels belonging to the same class at the same position in adjacent slices and contrastively decrease the similarity between voxels from different classes.
• **EMBL** (Heidelberg)^9^: A 5-level 3D U-Net [44]. The network predicts foreground probabilities and long-range affinity maps [40], specifically targeting pixel distances of 1, 3, and 9 along the $x$- and $y$-axes, and 1, 2, and 3 along the $z$-axis, taking into account the dataset’s anisotropy. For the same reason, no pooling is performed across the $z$-axis in the first two pooling layers of the 3D U-Net. The network is trained using the Dice score as the loss function. For obtaining an instance segmentation based on the foreground and affinity predictions, Mutex Watershed [49] is applied in parallel on the predictions of subvolumes of the entire dataset. To segment only mitochondria, the segmentation algorithm is applied solely to the foreground mask obtained by thresholding the foreground probability predictions at 0.5. Finally, the whole-volume instance segmentation is obtained by solving a Multicut clustering problem [50].
• **CEM-PDL** (NIH)^10^: A Panoptic-DeepLab model [51] with a ResNet50 [52] backbone is trained to perform instance segmentation in 2D slices. More specifically, the model has three outputs: semantic masks, instance centers, and instance center regressions (offset from each pixel to its corresponding center). Instances are obtained by simple post-processing (assigning each pixel to the closest predicted center). The backbone network uses weights pre-trained on CEM500K [53], a large dataset of EM images. Training is performed on patches of size $512 \times 512$ and the inference is applied to the full-size image ( $4096 \times 4096$). Several post-processing methods are used including $z$-filtering, 2D watershed to split false mergers, and the Hungarian algorithm [54] and the Intersection-over-Area merging strategy to merge false splits. This method has been further developed since submission to the MitoEM challenge into an open-source model called MitoNet [34].
• **FCI** (London)^11^ [35]: Four separate convolutional neural networks were trained to predict mitochondria binary masks in MitoEM-H, mitochondria boundaries in MitoEM-H, mitochondria binary masks in MitoEM-R, and mitochondria boundaries in MitoEM-R, respectively. All networks share a common architecture based on a 5-level 3D U-Net [44] with Inception-like blocks [55], 32 initial filters and a dropout rate of 0.3. The input size for the networks is $256\times 256\times 12$ and the loss function used was a smoothed Dice coefficient (or F-measure). Weights were initialized using a nuclear envelope segmentation model trained on crowd-sourced citizen science annotations [56]. The training sets of MitoEM-R and MitoEM-H were both divided into 16 equally sized stacks. Due to memory constraints, one training stack was presented per epoch. An initial model was trained to predict binary masks on both human and rat data, which was then fine-tuned on MitoEM-R and subsequently on MitoEM-H. The final weights from the MitoEM-R binary mask model served as the initial weights for the MitoEM-R boundary prediction model, and the weights from this model were used as initialization for the model predicting boundaries in MitoEM-H. To improve boundary predictions, the team combined predictions from all three views of the volumes after reslicing the data in the $xz$ and $yz$ planes and interpolating the $z$-scale from 30nm to 10nm, resulting in a voxel size of $8\times 8\times 10$nm [56]. Individual instances were then extracted using marker-controlled watershed [37] after creating seeds by subtracting the boundary masks from the binary masks.
• **ABCS** (FNL)^12^: The ABCS team trained two simplified 3D versions of the original U-Net architecture [36] to simulate different fields of view. The first network had an input size of $128\times 128\times 64$, and the second network had an input size of $256\times 256\times 64$. Each $4096\times 4096\times 1000$ volume was vertically split into four quadrants, resulting in four quadrants of size $1024\times 1024\times 500$ as sub-volumes for four-fold cross-validation training. During each fold of the cross-validation training, three out of the four quadrants were used as the training set, and the remaining quadrant was used as the validation set. Training and validation samples were randomly extracted from the corresponding quadrants at runtime and fed to the GPU. To account for boundary conditions, the original volumes were padded with blank pixels along all three axes. Basic data augmentation techniques, including flipping in all three axes, were applied during training. During inference, ensemble prediction with patch overlap was performed. Blank paddings were added to the two testing volumes as required by the sample extraction process. The combined segmentation outputs from both trained networks showed slightly better performance compared to the individual networks.
• **H2RNet** (Zurich)^13^: The H2RNet method is a hybrid instance segmentation approach that combines 2D and 3D processing. Initially, the method performs a segmentation on individual 2D slices of the volume using a modified HRNet [57] network. The HRNet has two heads, one for predicting the energy surface and the other for estimating the curvature of mitochondria in each 2D slice. The outputs of both heads are fused to obtain the final prediction. The input size of the network is set to $256\times 256$, but during inference, a patch size of $1024\times 1024$ is used to minimize border effects or artifacts from reassembled image crops. For training, a weighted mean-square-error loss function is employed. The weights are determined based on the frequency of a given value for the energy head, and bending loss [58] is used to compute the weights for the contour head. Unlike some other methods, H2RNet does not require watershed post-processing in 2D, as a cut-off from the learned surface energy is used as a hyper-parameter in the prediction. Due to computational limitations, the 2D predictions are downsampled before applying marker-controlled watershed [37] in 3D to connect regions across sections. The connected regions are then upsampled using nearest-neighbor interpolation.

^6^M. Li, C. Chen, Z. Xiong.^7^R. Xin, H. Liu, H. Chen.^8^Z. Li, J. Zhao, X. Chen.^9^C. Pape.^10^R. Conrad.^11^L. Nightingale, J. de Folter, M. L. Jones.^12^Y. Liu, D. Ziaei.^13^S. Huschauer.

## IV. Hits and Misses of Current Evaluation Metrics

Instead of using a single set of metrics, as it is common practice in the literature of 3D instance segmentation of biomedical images, we decided to perform an in-depth analysis of the most commonly used metrics to create a compact and informative error report to debug 3D instance segmentation methods. Moreover, this analysis allowed us to decide on the optimal metric to finally base the challenge ranking on.

### A. State-of-the-Art 3D Instance Segmentation Metrics

There are three commonly used sets of metrics for the 3D instance segmentation task: AP-based, matching-based, and association-based.

#### 1) AP-Based Metric:

The AP metric [12] relies on the calculation of other metrics such as IoU, precision, and recall. Let an *instance* be set of pixels/voxels belonging to an object, then the IoU measures the overlap between two instances ( $A$, $B$) and can be calculated as

\begin{equation*} IoU(A,B)=\frac {\vert A \cap B\vert }{\vert A\cup B\vert } \tag{1}\end{equation*}

where $\vert.\vert $ denotes the number of pixels (in 2D) or voxels (in 3D). Precision and recall are then defined as

\begin{align*} precision&=\frac {TP}{TP+FP}, \tag{2}\\ recall&=\frac {TP}{TP+FN} \tag{3}\end{align*}

where the true positive ( $TP$), false positive ( $FP$), and false negative ( $FN$) values depend on a predefined IoU threshold value (to consider two overlapping instances the same) and a probability confidence threshold (to consider the instance a mitochondrion). More specifically, a predicted instance is considered a TP if its IoU value with a ground truth instance is larger than the IoU threshold value, otherwise it is considered a FP. Moreover, ground truth instances without matching predictions are considered FN. For a fixed IoU threshold, a precision-recall curve can be created for a set of different confidence threshold values. The AP is the area under the precision-recall curve:

\begin{equation*} AP = \int _{0}^{1} p(r) \,dr \tag{4}\end{equation*}

where $p$ is precision and $r$ recall. The trade-off between precision and recall will decrease the precision-recall curve monotonically, as increasing one will decrease the other. Nevertheless, this rule does not occur consistently, resulting in a zigzag pattern. Henceforth, precision at each recall level $r$ is interpolated by taking the maximum precision when the corresponding recall exceeds $r$:

\begin{equation*} p_{interp}(r) = \max \limits _{\tilde {r}:\tilde {r}\geq r} p(\tilde {r}) \tag{5}\end{equation*}

where $p(\tilde {r})$ is the measured precision at recall $\tilde {r}$. Then, the AP is commonly approximated as the mean precision ( $p$) at a set of eleven equally spaced recall ( $r$) levels (from 0.0 to 1.0 with 0.1 increments):

\begin{equation*} AP = \frac {1}{11} \sum _{r\in \{0,0.1,\ldots,1\}} p_{interp}(r) \tag{6}\end{equation*}

In the present scenario, we employ AP-75, a metric that quantifies the AP computed at a threshold of 75% of the minimum IoU required to classify a detection as a TP (following [14]), otherwise the ground truth instance is considered a FN and the prediction a FP. In this manner, the IoU is calculated at pixel level but whereas a TP, FP and FN is defined at instance level.

A drawback of the AP metric is that it requires sorting predictions by confidence, which is not provided by most bottom-up segmentation approaches. Wei et al. [14] heuristically used the instance size as the prediction confidence, which can lead to undesirable biases for method ranking.

#### 2) Matching-Based Metrics:

Metrics based on matching focus on quantifying correctly predicted instances, transforming instance segmentation results into an object detection framework. In this paradigm, the emphasis shifts from uniquely labeled instances to detecting the presence or absence of instances. This transformation is achieved by establishing a criterion for instance overlap, commonly measured through IoU. Unlike traditional segmentation evaluations that rely on nuanced pixel-level overlaps, this approach simplifies assessment by classifying instances as successful (TP) based on a predefined IoU threshold. This aligns with decision-making processes in detection problems, providing a streamlined and robust evaluation strategy.

These metrics can combine informative statistics, *i.e.*, TP/FP/FN, into a single value to rank the methods. More specifically, we use accuracy, which is defined as follows:

\begin{equation*} accuracy=\frac {TP}{TP+FP+FN} \tag{7}\end{equation*}

To decide which predicted instance corresponds to a ground truth instance we make the following definitions. Let us assume we have two sets: one consisting of the predicted instances, denoted by $\mathcal {P}$, and another set containing the ground truth instances, denoted by $\mathcal {G}$. The mathematical representation of the scenario can be expressed as follows:

\begin{equation*} \mathcal {P} = \{p_{1}, p_{2}, p_{3}, \ldots, p_{n}\} \tag{8}\end{equation*}

where $p_{i}$ are the predicted instances and $n$ is the number of instances in the predicted set.

\begin{equation*} \mathcal {G} = \{g_{1}, g_{2}, g_{3}, \ldots, g_{m}\} \tag{9}\end{equation*}

where $g_{j}$ are the ground truth instances and $m$ is the number of instances in the ground truth set.

Following previous work [9], [10], in order to decide the optimal assignment of predicted and ground truth instances, we use the Hungarian algorithm [54] whereby an instance cannot be assigned to multiple ground-truth instances (and vice versa). In our case, the optimal assignment is given by the following cost

\begin{equation*} min \sum _{i} \sum _{j} C_{i,j} X_{i,j} \tag{10}\end{equation*}

where $X$ is a boolean matrix, wherein an element $X_{i,j}$ is true if and only if row $i$ is assigned to column $j$, and $C$ is the cost matrix, defined as:

\begin{equation*} C(i,j) = \frac {-(IoU(g_{j},p_{i}) >= T) - IoU(g_{j},p_{i})}{(2*N)} \tag{11}\end{equation*}

where $T$ is the threshold 0.75, as in AP-75, and $N$ is the number of assignments (minimum between the number of predicted instances and the number of ground truth instances).

#### 3) Association-Based Metrics:

Many segmentation methods need to set hyper-parameters to control the ratio between false-split and false-merge errors. Thus, a pie chart displaying the proportion of different types of segmentation association error [15] is critical for a more interpretable result understanding. Using the previously computed IoU values as in the matching-based metrics, the set of pairs of associated regions between $p_{i}$ and $g_{j}$ can be defined as follows:

\begin{equation*} \mathcal {A} = \{(p_{i}, g_{j}) \mid IoU(p_{i}, g_{j}) > 0, p_{i} \in \mathcal {P}, g_{j} \in \mathcal {G}\} \tag{12}\end{equation*}

Let us define the two sets $\mathcal {A}(g_{j})=\{p_{i}\vert (p_{i},g_{j})\in \mathcal {A}\}$ and $\mathcal {A}'(p_{i})=\{g_{j}\vert (p_{i},g_{j})\in \mathcal {A}\}$ corresponding to the ground truth instances $g_{j}$ associated with predicted instances $p_{i}$ and predicted instances $p_{i}$ associated with ground truth instances $g_{j}$, respectively. Then, different cases of resulting reciprocal mapping are considered:

• *One-to-one*, when there is an exact match between $g_{j}$ and $p_{i}$: $\mathcal {A}(g_{j})=\{p_{i}\}\,\,and\,\,\mathcal {A}'(p_{i})=\{g_{j}\}$.
• *Over-segmentation*, when one instance in the ground truth is divided into two or more in the prediction: $\vert \mathcal {A}(g_{j})\vert >1\,\,and\,\,\forall {p_{i}}\in \mathcal {A}(g_{j}), \mathcal {A}'(p_{i})=\{g_{j}\}$.
• *Under-segmentation*, when two or more instances in the ground truth are merged in the prediction: $\vert \mathcal {A}'(p_{i})\vert >1\,\,and\,\,\forall {g_{j}}\in \mathcal {A}'(p_{i}), \mathcal {A}(g_{j})=\{p_{i}\}$.
• *Missing*, for instances of the ground truth that are not captured in the prediction: $\mathcal {A}(g_{j})=\emptyset $.
• *Background*, for instances associated with the background, i.e. false positives: $\mathcal {A}'(p_{i})=\emptyset $.
• *Many-to-many*, all other cases.

In summary, *background* associations are typically expressed as a percentage of the total number of predicted instances, whereas the remaining associations are expressed as a percentage of the total number of ground truth instances. Consequently, the cumulative percentage of these non-*background* associations amounts to 100%.

### B. Discussion on 3D Instance Segmentation Metrics

**Fig. 4.**
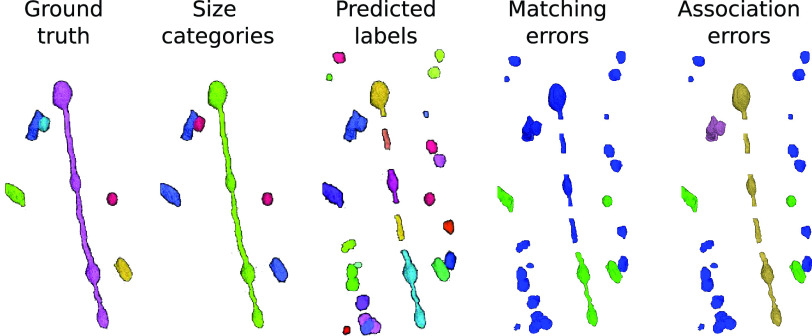
Synthetic example of mitochondria instance segmentation. Left to right: ground truth instances, same instances color-coded by size (small in red, medium in blue and large in green), model prediction, matching errors (FP in blue and TP in green) and association errors (*one-to-one* in green, *background* in blue, *over-segmentation* in yellow and *under-segmentation* in magenta).

To better understand the pros and cons of each metric, we created a toy example with ground truth mitochondria instances of different sizes and realistic model predictions (see Fig. 4). The ground truth volume contains only six instances: one large (MOAS type), three medium, and two small mitochondria based on their cable lengths. The prediction presents an *over-segmentation* of small and medium instances, a merger of two mitochondria, and several split errors in the MOAS. The corresponding AP-75, association, and accuracy values are shown in Table II.

#### 1) AP-75 Overvalues Small-Size Instances:

In our ISBI challenge, we developed an efficient implementation of AP-75 for 3D volumes. Due to the lack of confidence prediction for each instance, we sorted mitochondria instances by size, resulting in small instances having the lowest confidence values. Therefore, when a small instance is merged with a medium one in the prediction, the small instance is considered an FN. Additionally, the large instance in the ground truth is split into several instances that do not reach the minimum IoU of 75% with the ground truth, so most of those instances are considered as medium FPs. This means the large mitochondrion is only matched with the blue instance that represents its bottom part (since it is the largest among all pieces). Although the prediction contains several small FPs, as well as more small and medium FPs considering the rest of the MOAS pieces not matched with it (e.g. all but the blue instance), the AP-75 values for small instances are still high. Note that other drawbacks of the AP metric were discussed in recent papers [59] from different angles.

#### 2) Accuracy Metric Provides a Good Overall Evaluation:

As shown in Table II, the association metrics are useful for understanding the fate of the ground truth instances in the prediction but do not provide information on the overlap between the prediction and the ground truth. On the other hand, the matching metrics do provide this information by considering a prediction as a TP if the IoU with ground truth is greater than 75%. However, the association metrics have multiple values, rather than just a single one, which complicates the direct comparison of the performance of different methods. For example, it is not clear whether a low *under-segmentation* rate is better or worse than a low *over-segmentation* rate, or whether *many-to-many* is worse than the previous two. These questions depend on the specific task at hand. Therefore, it is useful to have a single number, such as accuracy, to enable easy comparison of the performance of different models. In the toy example, there are many small FPs in the prediction, as previously mentioned, which results in low values for all matching metrics except recall. For medium instances, only the one merged with the small instance is not considered a TP due to its low IoU (< 0.75).

#### 3) Association Metrics Provide a Detailed Breakdown of Errors:

Examining the association metrics helps us to understand where and how the prediction fails. A *missing* value of zero in all cases indicates that all ground truth instances have been captured by the prediction. More specifically, out of the two small mitochondria in the ground truth, one has been correctly predicted and is labeled as *one-to-one*. The other one was merged with a medium mitochondrion, resulting in both small and medium being labeled as *under-segmentation*. The remaining three medium mitochondria are also labeled as *one-to-one*. Also, the ground truth MOAS that was divided into medium-sized pieces in the prediction is labeled as *over-segmentation*.

## V. Analysis of Current Progress on MitoEM

In this section, we leverage on the evaluation metrics defined in the previous section to analyze in detail the performance of the participant and baseline methods in the challenge.

### A. Overall Performance

#### 1) Matching-Based Evaluation:

The matching metric values corresponding to the top submissions of all methods on the leaderboard are presented in Table III. The IIPPR method demonstrates superior performance compared to VIDAR in most cases, except for recall. This trend is also observed in other methods, where high recall comes at the cost of precision. For instance, U3D-BC, VGG, and U2D-BC exhibit much higher recall than precision values, indicating a larger number of false positives. A detailed breakdown analysis of matching-based metrics for each instance category (small, medium, and large) can be found in Table VI in the Appendix. Notably, all methods exhibit better segmentation of small and medium mitochondria compared to large mitochondria in both MitoEM-R and MitoEM-H datasets. Furthermore, it is evident that segmenting large mitochondria in MitoEM-H is more challenging than in MitoEM-R, as confirmed by visual inspection in Fig. 2.

**TABLE VI:** Matching-Based Metrics of All Methods on the MitoEM Challenge Leaderboard Per Category. The Baseline Methods From the Challenge Organizers (Marked With *) are Shown but Not Included in the Ranking. Bold and Underlined Numbers Denote the 1st and 2nd Scores, Respectively

Method	Accuracy Rank		MitoEM-H			MitoEM-R		
		Category	Precision $\uparrow $	Recall $\uparrow $	Accuracy $\uparrow $	Precision $\uparrow $	Recall $\uparrow $	Accuracy $\uparrow $
IIPPR	1	large	0.148	0.543	0.132	0.397	0.779	0.357
		medium	0.930	0.934	0.872	0.969	0.967	0.938
		small	0.811	0.910	0.750	0.753	0.938	0.717
VIDAR	2	large	0.172	0.549	0.151	0.353	0.794	0.323
		medium	0.916	0.946	0.870	0.961	0.974	0.936
		small	0.758	0.924	0.713	0.362	0.933	0.353
U3D-BC*		large	0.131	0.537	0.118	0.268	0.763	0.247
		medium	0.890	0.933	0.836	0.936	0.934	0.878
		small	0.662	0.917	0.625	0.479	0.910	0.457
EMBL	3	large	0.108	0.549	0.099	0.231	0.725	0.212
		medium	0.853	0.905	0.783	0.786	0.940	0.748
		small	0.756	0.871	0.680	0.629	0.878	0.578
VGG	4	large	0.109	0.506	0.099	0.323	0.708	0.285
		medium	0.884	0.915	0.818	0.938	0.927	0.873
		small	0.603	0.922	0.574	0.494	0.919	0.473
CEM-PDL	5	large	0.164	0.427	0.134	0.232	0.519	0.191
		medium	0.834	0.849	0.727	0.900	0.901	0.819
		small	0.711	0.768	0.585	0.652	0.878	0.597
FCI	6	large	0.167	0.470	0.141	0.229	0.540	0.191
		medium	0.821	0.831	0.703	0.833	0.813	0.699
		small	0.733	0.709	0.564	0.624	0.741	0.513
H2RNet	7	large	0.117	0.341	0.096	0.214	0.515	0.178
		medium	0.580	0.692	0.461	0.865	0.852	0.752
		small	0.734	0.715	0.568	0.733	0.812	0.626
ABCS	8	large	0.134	0.537	0.120	0.381	0.548	0.290
		medium	0.776	0.787	0.641	0.808	0.684	0.588
		small	0.592	0.758	0.498	0.556	0.784	0.482
U2D-BC*		large	0.166	0.530	0.145	0.213	0.708	0.196
		medium	0.865	0.937	0.818	0.906	0.930	0.848
		small	0.328	0.929	0.320	0.137	0.936	0.135

#### 2) Association-Based Evaluation:

Table IV presents the association metric values for all ranked methods, including our own baseline methods. The absolute numbers of association types per instance category for each participant method is illustrated in Fig. 10 in the Appendix. In both human and rat tissues, the IIPPR method achieves the highest *one-to-one* value, representing the percentage of correctly associated ground truth instances. Furthermore, as shown in Table IV, IIPPR exhibits very low *over-segmentation* values, in contrast to other methods such as U3D-BC, EMBL, or VGG. This aligns with their previously observed high recall values (Table III), which are a result of a larger number of false positive instances. However, accurately assessing the methods solely based on the percentage of correctly assigned instances (*one-to-one* value) is insufficient, as it can be accompanied by a high number of *background* associations, as observed in the VGG or U2D-BC methods.

**Fig. 5.**
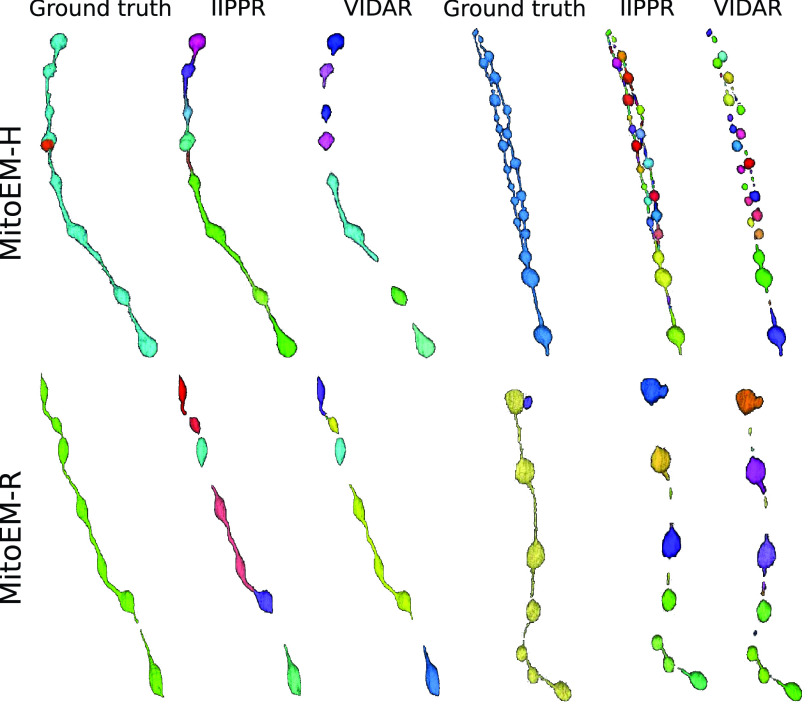
3D visualization of MOAS instances for error inspection. We show the ground truth and segmentation results from the two top-performing models (IIPPR and VIDAR) in one MOAS instance per dataset. Different colors represent different instances.

**Fig. 6.**
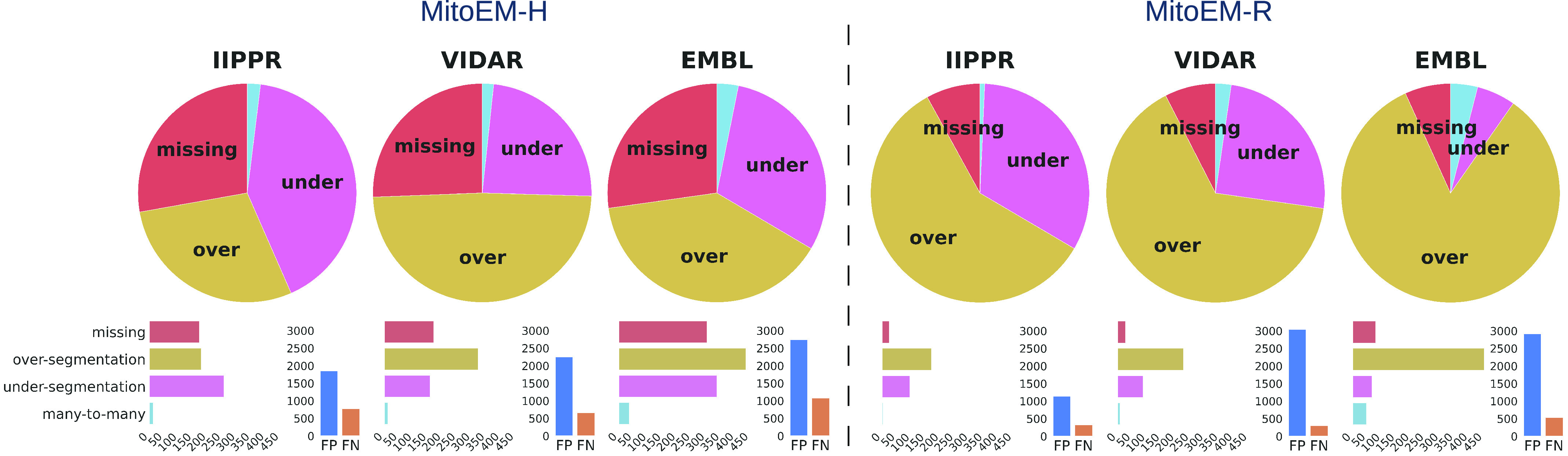
Summary of association errors in MitoEM for the top three methods: IIPPR, VIDAR, and EMBL. The pie charts illustrate the proportions of association errors relative to the ground truth instances. The bar plots below depict the absolute magnitudes of the association errors (left), and the total number of false positives (FP) and false negatives (FN) for each method (right).

**Fig. 7.**
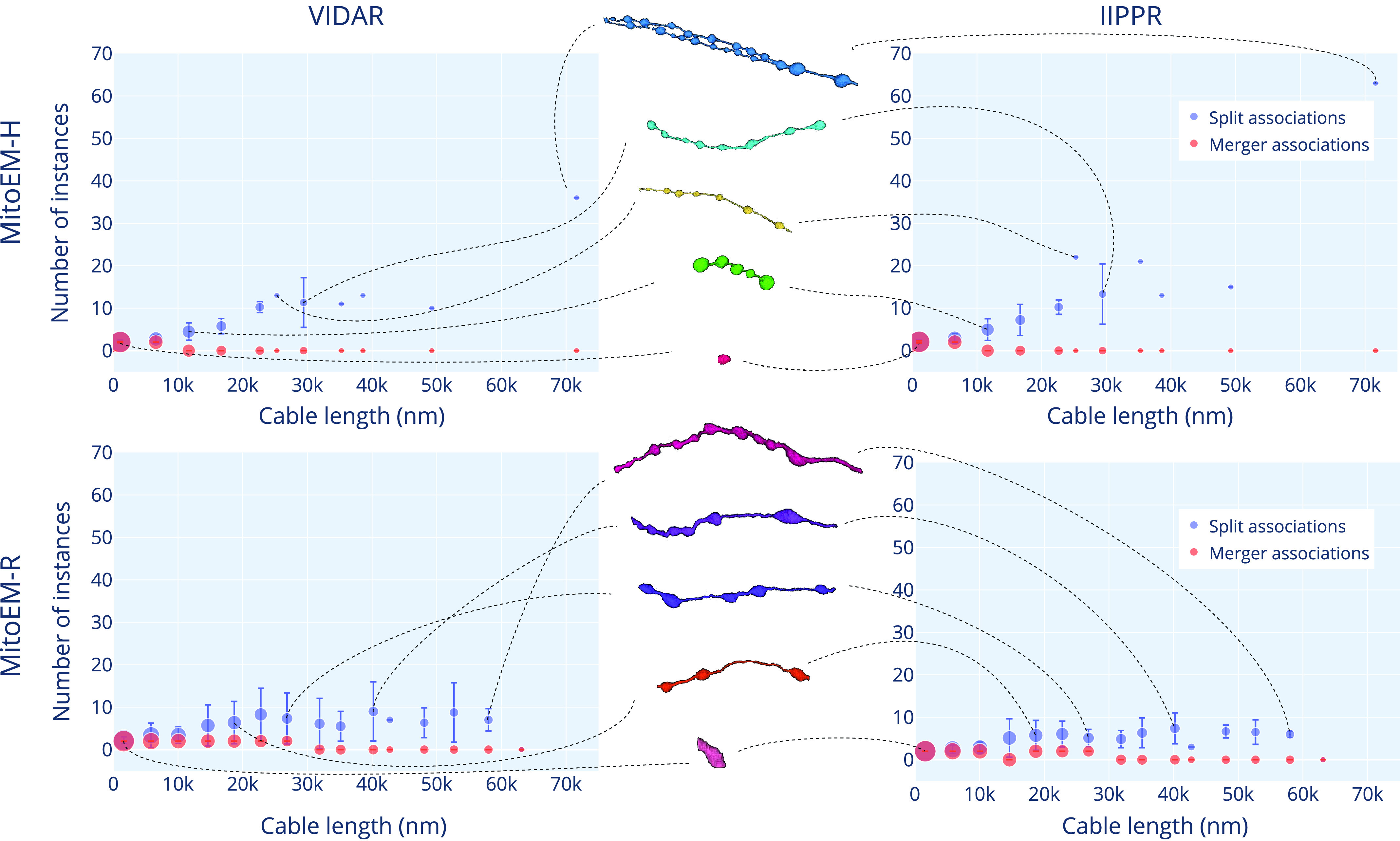
Split vs. merger instances distribution for the top two methods (VIDAR and IIPPR) on the MitoEM-H (top) and MitoEM-R (bottom) test sets. Each data point represents the number of instances for mitochondria of different lengths, with vertical lines indicating the standard deviation. The size of each data point is proportional to the number of instances within that length bin. Representative instances of various cable lengths are displayed in the middle and connected to their respective bins by dashed lines. The skeleton length is evenly divided into 15 bins and measured in the ground truth instances.

**Fig. 8.**
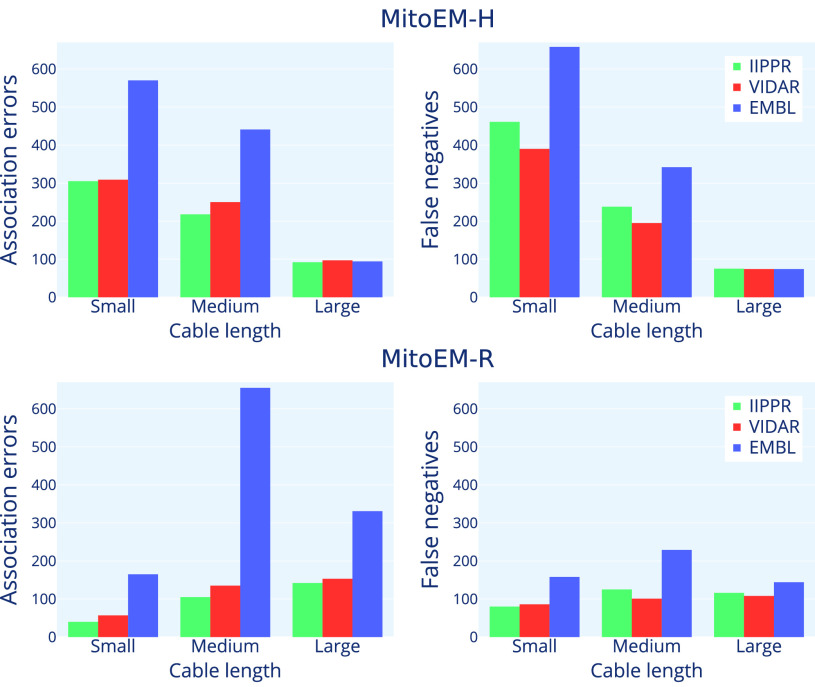
Summary of the absolute number of error types per instance category for the three top-performing methods. The errors shown include cumulative association (i.e., *missing*, *over-segmentation*, *under-segmentation* and *many-to-many*) errors (on the left) and false negatives (on the right) for each method.

**Fig. 9.**
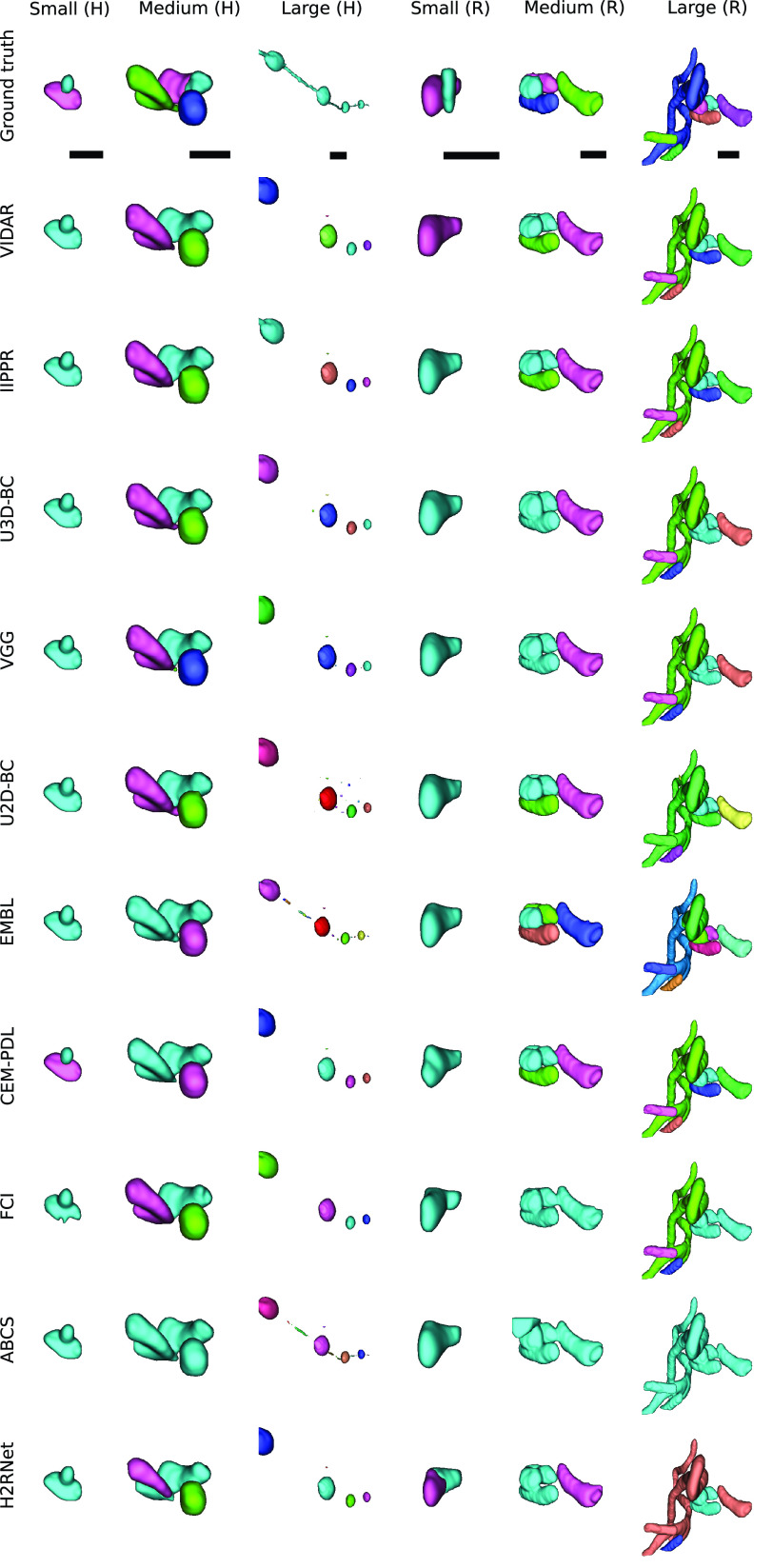
Some examples of common segmentation errors by the analyzed methods in small, medium and large mitochondria of MitoEM-H and MitoEM-R tissue from the test set. Every instance is given a unique color. The scale bar represents $0.5~\mu \text{m}$.

**Fig. 10.**
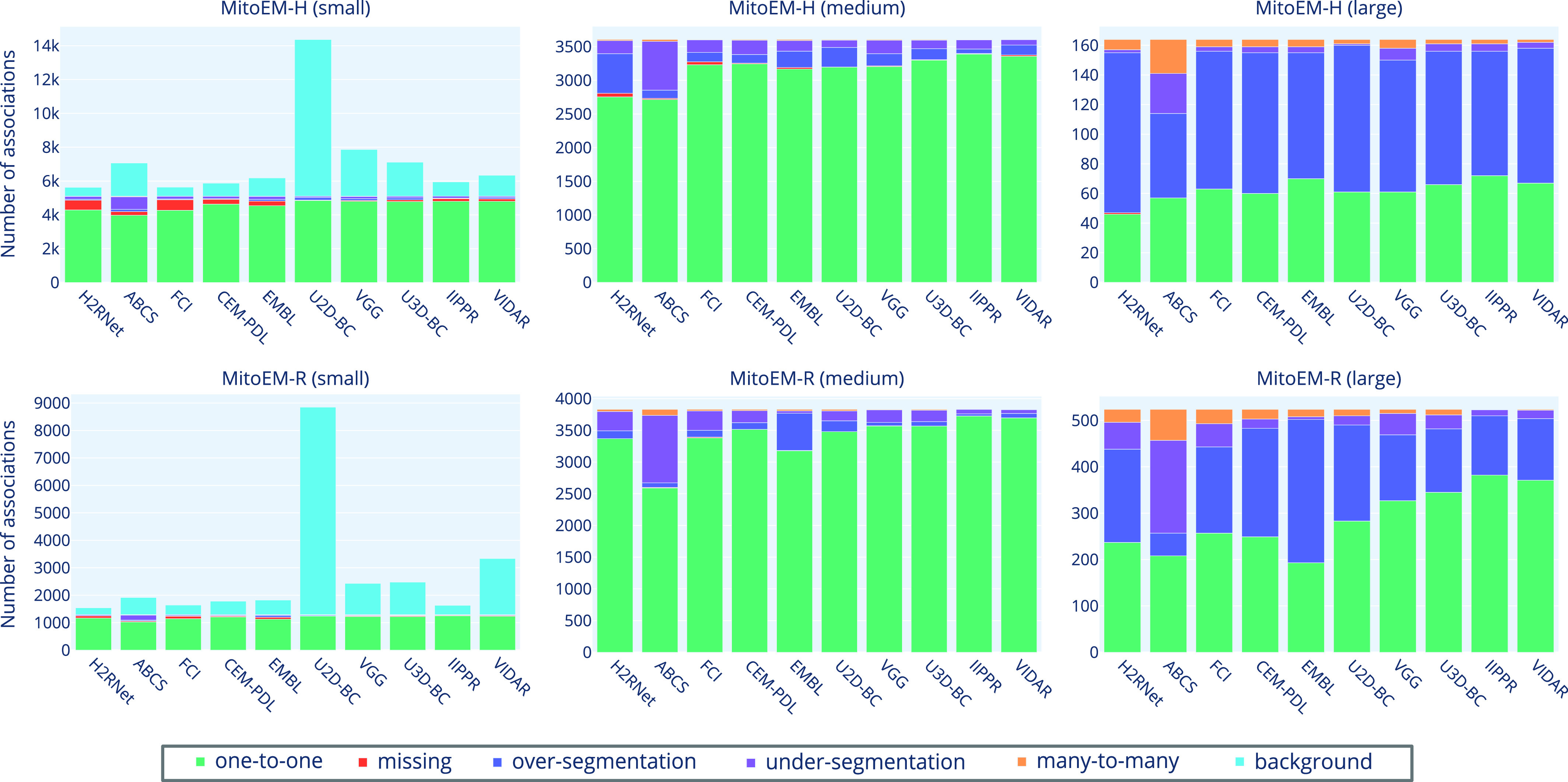
Distribution of types of associations for all participant methods on the MitoEM-H (top) and MitoEM-R (bottom) test sets for small (left), medium (center) and large (right) mitochondria. The methods are ordered from left to right by lowest-to-highest value of AP-75.

To gain further insights into the types of association errors made by the top three methods (IIPPR, VIDAR, and EMBL), we present two analyses in Fig. 6: (1) an overview of the errors relative to the ground truth instances, and (2) their absolute magnitudes for method comparison. Generally, Fig. 6 reveals that the relative magnitude of missing instances is similar among the top three methods for both tissues. However, the absolute magnitudes indicate better performance for IIPPR and VIDAR compared to EMBL. The top methods tend to exhibit *over-segmentation* rather than *under-segmentation* (except for IIPPR in human tissue). This highlights the challenges faced by these methods in accurately segmenting the most difficult instances in MitoEM, particularly the MOAS-type mitochondria. This observation is also supported by the high number of *over-segmentation* associations for large mitochondria, as illustrated in Fig. 10 in the Appendix. Some visual examples of *over-segmentation* of MOAS specifically for the top two methods are shown in Fig. 5. Additionally, examples of common errors for all methods in all mitochondria categories are shown in Fig. 9 in the Appendix.

### B. Comparison Across Skeleton Length

Overall, the complexity of mitochondria is influenced by the length of the skeleton. The length of the skeleton can vary depending on the type and size of the cell in which the mitochondria are located. Based on the overall performance of the methods, we have identified a clear issue of *over-segmentation* of large mitochondria in both tissues. However, we have not yet considered the number of instances that the splitting or merging of instances involve. Therefore, it is important to compare the number of instances associated with *over-segmentation*, *under-segmentation*, and *many-to-many* associations to determine which type of error has the most significant impact.

Let us define the sets $\mathcal {OS(\mathcal {A})}$, $\mathcal {US(\mathcal {A})}$, and $\mathcal {MM(\mathcal {A})}$ corresponding to the over-segmentation, under-segmentation and many-to-many associations in $\mathcal {A}$, respectively. We then define the subset $\mathcal {S}$ of association splits as

\begin{align*} \mathcal {S} &= \{(p_{i}, g_{j}) \mid (p_{i}, g_{j}) \in \mathcal {OS(\mathcal {A})}~or \\ (p_{i}, g_{j}) &\in \mathcal {MM(\mathcal {A})}, \vert \mathcal {A}'(p_{i})\vert \geq \vert \mathcal {A}(g_{j})\vert \}. \tag{13}\end{align*}

Similarly, we define the subset $\mathcal {M}$ of association mergers as:

\begin{align*} \mathcal {M} &= \{(p_{i}, g_{j}) \mid (p_{i}, g_{j}) \in \mathcal {US(\mathcal {A})}~or \\ (p_{i}, g_{j}) &\in \mathcal {MM(\mathcal {A})}, \vert \mathcal {A}'(p_{i})\vert < \vert \mathcal {A}(g_{j})\vert \}. \tag{14}\end{align*}

To compare the number of instances in each subset, we use $\vert \vert \mathcal {S}\vert \vert _{p}$ and $\vert \vert \mathcal {M}\vert \vert _{g}$, where $\vert \vert.\vert \vert _{p}$ is the number of elements related to prediction instances in the subset (i.e. $\vert \mathcal {A'}(p_{i})\vert $) and $\vert \vert.\vert \vert _{g}$ is the number of elements related to ground truth instances in the subset (i.e. $\vert \mathcal {A}(g_{j})\vert $). In Fig. 7, we present the number of *split* and *merger* instances as a function of the cable length (measured in the ground truth instances) for both MitoEM-H and MitoEM-R, focusing on the results of the top two methods (VIDAR and IIPPR). It can be observed that, in all cases, the number of instances associated with splits tends to increase with cable length, while the number of instances associated with mergers remains relatively low across different lengths. This observed trend can be attributed to the presence of MOAS-type mitochondria, where larger structures tend to consist of a higher quantity of smaller constituent elements. Furthermore, it is worth noting that the results for MOAS in human tissue exhibit a greater number of splits compared to rat tissue. This discrepancy is likely due to the thicker connections present in rat MOAS within this specific dataset, as depicted in the middle of Fig. 7, which make them easier to segment in 3D. However, it is important to acknowledge that these differences in size may not be representative of all humans and rats. Therefore, further investigation and a larger sample size would be necessary to validate and establish reference ranges for mitochondrial sizes in these species.

Fig. 8 provides a detailed analysis to identify the types of mitochondria that exhibit the highest failure rates for the top three methods. When considering the absolute number of cumulative association errors (*missing*, *over-segmentation*, *under-segmentation*, and *many-to-many*), the results align with the ranking presented in Table III, with IIPPR performing the best, followed by VIDAR, and finally EMBL. However, in terms of false negatives, VIDAR outperforms IIPPR. This finding is consistent with the results discussed in Section V-A, which indicate that VIDAR is capable of identifying more instances, albeit at the expense of higher false positive rates and lower precision.

## VI. Discussion on Remaining Challenges

Despite the notable improvements achieved during the competition, there are still several challenges that the research community needs to address.

### A. Model Challenge

In the current setting of full-supervised learning with a 40-10-50% data split, the IIPPR method serves as a strong baseline, achieving an overall accuracy score of 0.770. However, for practical deployment on recent petabyte-scale datasets [17], instance segmentation methods must achieve even higher accuracy to make the proofreading process feasible at scale (preferably above 0.9 based on our own proofreading experience). In addition to the inherent challenges posed by the datasets, such as complex geometries and crowded instances, there remains an open challenge in accurately segmenting “large” instances, particularly MOAS instances with super-thin connections, as they often result in *over-segmentation*. To address this issue, the VIDAR team at USTC’s lab has proposed the use of knowledge distillation training [60] as a potential solution. Furthermore, while the majority of methods show significantly better performance for “small” instances compared to “large” instances in terms of accuracy, all methods demonstrate much better results for “medium” instances (refer to Table III in the Appendix). This observation holds true even when the dataset contains a larger number of “small” instances than “medium” instances, as observed in the case of MitoEM-H. This indicates that the current architectures are more suitable for a specific length of mitochondria, leaving room for improvement in designing methods that can handle various lengths effectively.

### B. Limited Label Challenge

While the challenge was conducted within a fully supervised learning framework, it is important to acknowledge that in practical scenarios, the availability of labeled data is often limited to around 5-10% of the entire volume. Therefore, it is crucial to develop data-efficient methods that can achieve high accuracy with a limited amount of annotation. This includes exploring new data augmentation techniques [61], investigating unsupervised learning approaches [62], exploring semi-supervised learning methods, and leveraging active learning strategies. By addressing the limited label challenge, we can enable the development of models that effectively utilize a small amount of labeled data to achieve accurate segmentation results. We believe that our MitoEM dataset can also serve as a valuable resource for simulating and evaluating these learning settings.

### C. Proofreading Challenge

Regarding the suitability of a scoring system based on accuracy, one should assess the purpose of the segmentation result and its subsequent processing. In particular, for large datasets such as MitoEM, the current strategy assumes a proofreading phase after automatic segmentation. In that sense, a metric that does not penalize false positive predictions as much as false negative ones may be the most appropriate. In fact, eliminating false positives is proven much faster than correcting false negatives when proofreading 3D instances [63]. In a more general framework, the association and matching metrics provided by our in-depth analysis help us complete the big picture in terms of evaluation.

## VII. Conclusion

In this paper, we present the results of the ISBI 2021 challenge on MitoEM, the first large-scale instance mitochondria segmentation challenge that thoroughly benchmarks state-of-the-art methods in the field. To gain insight into the common errors of the proposed methods and identify current challenges that remain unresolved, we analyze the performance of the methods using various types of evaluation metrics.

The release of MitoEM had the dual goal of attracting new computer vision researchers to the problem of EM mitochondria segmentation and pushing the state of the art forward. We believe that the challenge was successful in this regard, as the participants improve over our own initial baseline methods. Furthermore, the competition received a very positive reaction from the community and had good attendance at its corresponding workshop at ISBI 2021.

After conducting a comprehensive analysis of the challenge results, we identified consistent annotation errors and addressed them by releasing an updated version of the ground truth labels (V2). Furthermore, through a thorough examination of the state-of-the-art evaluation metrics, we identified issues with the evaluation system based on the AP-75 metric and updated the challenge and method ranking using accuracy, which is a more robust metric that takes into account false negatives and *over-segmentations* more effectively. Nevertheless, the current accuracy values are still insufficient for fully automatic segmentation, therefore the challenge remains open for submissions.

Finally, we would like to highlight the potential of our large-scale annotated dataset for a wide range of applications beyond its original purpose. The dataset can be used for tasks such as deep feature pre-training, 3D shape analysis, and testing novel approaches including active learning or domain adaptation. The availability of this dataset provides valuable opportunities for researchers to explore new directions and tackle various challenges in the field of mitochondria segmentation.

As future work, we will consider expanding the MitoEM dataset to create new interations of the challenge using the newly proposed score system, and thus enhancing the limited generalizability of the results produced on only two EM datasets.

## Appendix

The original challenge leaderboard, which initially ranked the methods based on AP-75 performance, is presented in Table V. For a detailed breakdown analysis of matching-based metrics per mitochondria category, we refer to Table VI, which showcases the results of the top-performing submissions from both the participant and baseline methods.

Fig. 9 illustrates visual examples of common segmentation errors made by each participant method. The examples cover all mitochondria categories and tissues, allowing for a visual inspection of the errors made by different methods.

Additionally, to provide a comprehensive understanding of the associations per mitochondria category (small, medium, and large), we present the distribution of associations in Fig. 10 for the best submissions among all participant methods.
