# Supplementary material for: Current Progress and Challenges in Large-Scale 3D Mitochondria Instance Segmentation
Source: IEEE Trans Med Imaging. Author manuscript; Available in PMC 2024 Jan 19. (PMC10753957; doi:10.1109/TMI.2023.3320497)
Supplement: Appendix [file EMS191869-supplement-Appendix.pdf]

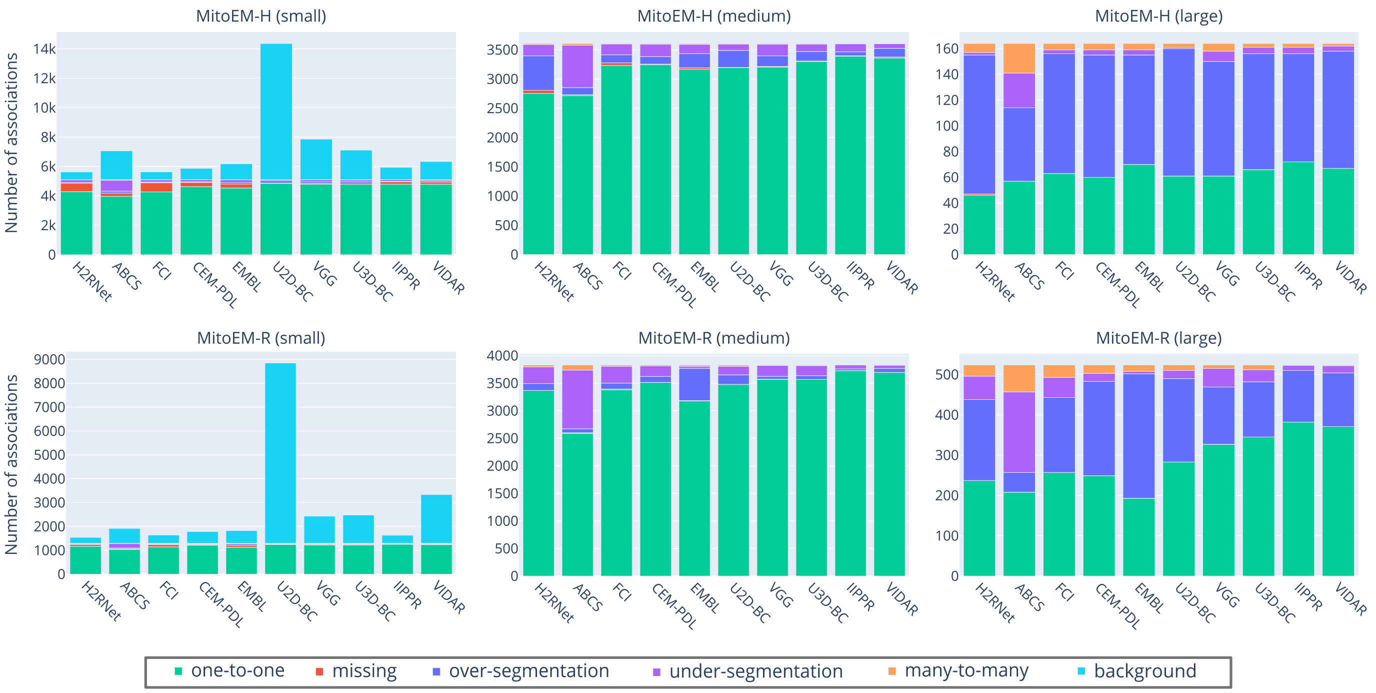

Fig. 10. Distribution of types of associations for all participant methods on the MitoEM-H (top) and MitoEM-R (bottom) test sets for small (left), medium (center) and large (right) mitochondria. The methods are ordered from left to right by lowest-to-highest value of AP-75.

methods. Furthermore, the competition received a very positive reaction from the community and had good attendance at its corresponding workshop at ISBI 2021.

After conducting a comprehensive analysis of the challenge results, we identified consistent annotation errors and addressed them by releasing an updated version of the ground truth labels (V2). Furthermore, through a thorough examination of the state-of-the-art evaluation metrics, we identified issues with the evaluation system based on the AP-75 metric and updated the challenge and method ranking using accuracy, which is a more robust metric that takes into account false negatives and *over-segmentations* more effectively. Nevertheless, the current accuracy values are still insufficient for fully automatic segmentation, therefore the challenge remains open for submissions.

Finally, we would like to highlight the potential of our large-scale annotated dataset for a wide range of applications beyond its original purpose. The dataset can be used for tasks such as deep feature pre-training, 3D shape analysis, and testing novel approaches including active learning or domain adaptation. The availability of this dataset provides valuable opportunities for researchers to explore new directions and tackle various challenges in the field of mitochondria segmentation.

As future work, we will consider expanding the MitoEM dataset to create new interations of the challenge using the newly proposed score system, and thus enhancing the limited generalizability of the results produced on only two EM datasets.

Appendix

The original challenge leaderboard, which initially ranked the methods based on AP-75 performance, is presented in Table V. For a detailed breakdown analysis of matching-based metrics per mitochondria category, we refer to Table VI, which showcases the results of the top-performing submissions from both the participant and baseline methods.

Fig. 9 illustrates visual examples of common segmentation errors made by each participant method. The examples cover all mitochondria categories and tissues, allowing for a visual inspection of the errors made by different methods.

Additionally, to provide a comprehensive understanding of the associations per mitochondria category (small, medium, and large), we present the distribution of associations in Fig. 10 for the best submissions among all participant methods.

Acknowledgment

The authors would like to thank the Grand Challenge Team for providing the platform that enables public access, challenge organization, and automatic evaluation.

Daniel Franco-Barranco is with the Department of Computer Science and Artificial Intelligence, University of the Basque Country (UPV/EHU), 20018 San Sebastián, Spain, and also with the Donostia International Physics Center (DIPC), 20018 San Sebastián, Spain (e-mail: daniel_franco001@ehu.eus).

Zudi Lin, Won-Dong Jang, and Hanspeter Pfister are with the Harvard John A. Paulson School of Engineering and Applied Sciences (SEAS), Harvard University, Allston, MA 02134 USA (e-mail: linzudi@gmail.com; wdjang@amazon.com; pfister@seas.harvard.edu).

Xueying Wang, Wenjie Yin, and Yutian Fan are with the Department of Molecular and Cellular Biology, Harvard University, Cambridge, MA 02138 USA (e-mail: snow.xwang@gmail.com; kelly.wjyin@gmail.com; timfan2002@gmail.com).

Qijia Shen is with the Wellcome Centre for Integrative Neuroimaging, FMRIB, Nuffield Department of Clinical Neurosciences, University of Oxford, OX3 9DU Oxford, U.K. (e-mail: qijia.shen@gtc.ox.ac.uk).
